# Rapid Evolution to Blast Crisis Associated with a Q252H *ABL1* Kinase Domain Mutation in e19a2 *BCR-ABL1* Chronic Myeloid Leukaemia

**DOI:** 10.1155/2013/490740

**Published:** 2013-09-16

**Authors:** Sarah L. McCarron, Karena Maher, Johanna Kelly, Mary F. Ryan, Stephen E. Langabeer

**Affiliations:** ^1^Cancer Molecular Diagnostics, Central Pathology Laboratory, St. James's Hospital, Dublin 8, Ireland; ^2^Department of Haematology, Waterford Regional Hospital, Waterford, Ireland; ^3^National Centre for Medical Genetics, Our Lady's Children's Hospital, Dublin 12, Ireland

## Abstract

A minority of chronic myeloid leukaemia (CML) patients express variant transcripts of which the e19a2 *BCR-ABL1* fusion is the most common. Instances of tyrosine kinase inhibitor (TKI) resistance in e19a2 *BCR-ABL1* CML patients have rarely been reported. A case of e19a2 *BCR-ABL1* CML is described in whom imatinib resistance, associated with a Q252H *ABL1* kinase domain mutation, became apparent soon after initiation of TKI therapy. The patient rapidly transformed to myeloid blast crisis (BC) with considerable bone marrow fibrosis and no significant molecular response to a second generation TKI. The clinical course was complicated by comorbidities with the patient rapidly succumbing to advanced disease. This scenario of Q252H-associated TKI resistance with rapid BC transformation has not been previously documented in e19a2 *BCR-ABL1* CML. This case highlights the considerable challenges remaining in the management of TKI-resistant BC CML, particularly in the elderly patient.

## 1. Introduction

The *BCR-ABL1* oncogene is the molecular hallmark and causative event of CML. Most CML patients express either e13a2 or e14a2 *BCR-ABL1* fusion transcripts, but approximately 5% of patients express variant transcripts that may involve fusion of alternative exons, insertions, or breakpoints within exons. Of these variant *BCR-ABL1* fusions, e19a2 is the most common. CML with e19a2 *BCR-ABL1*, that encodes a 230 kDa protein [1], was initially reported in neutrophilic CML with a relatively indolent clinical course [2] but has subsequently been reported in typical CML presenting in all phases [3–8].

Soon after the introduction of TKI therapy for CML, it became apparent that a significant proportion of patients are either primary refractory, display suboptimal responses, or acquire resistance to imatinib [9]. One of the most common causes of acquired resistance is the development of mutations within the *BCR-ABL1* kinase domain (KD) that prohibit effective binding of imatinib [10]. Greater than ninety of these mutations have been reported in imatinib resistant CML patients, the effects of the majority of which may be overcome by imatinib dose escalation or a second generation TKI [11]. Very few *BCR-ABL1* KD mutations have been reported in e19a2 *BCR-ABL1* CML [12–15] making an informed choice of subsequent TKI or alternative therapeutic approach problematic in the context of this transcript type.

Despite evidence suggesting that fewer patients transform to accelerated and blast crisis (BC) phases when treated with long term imatinib [16], transformation still occurs in a minority of CML patients. Second generation TKIs, allogeneic haematopoietic stem cell transplantation, and investigational agents have been shown to be of benefit [17], yet the outlook remains poor for a significant number, particularly those elderly patients with significant comorbidities.

## 2. Case Report

**Figure 1:**
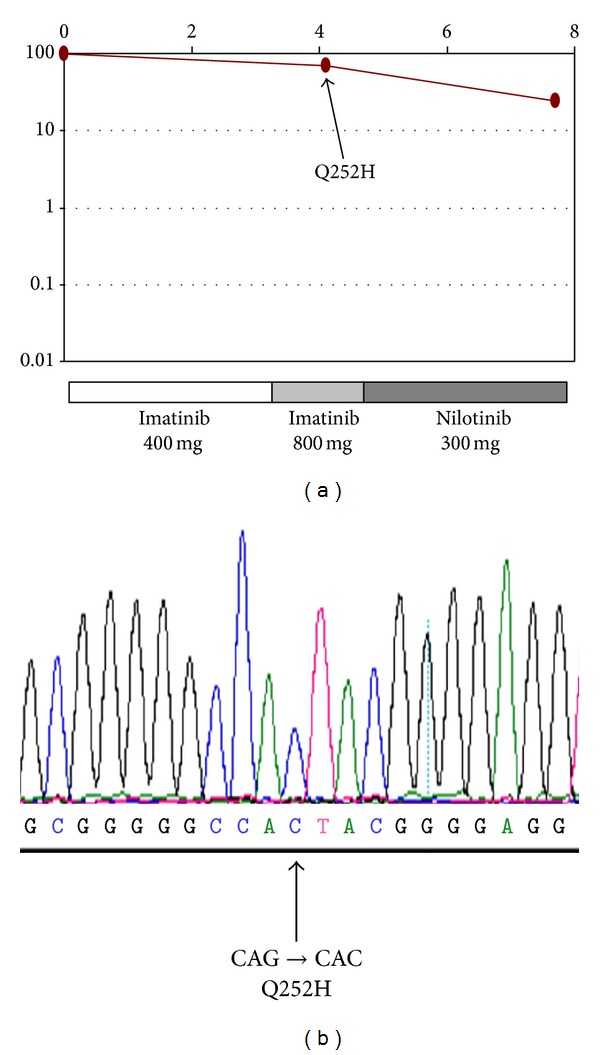
(a) qPCR of e19a2 *BCR-ABL1* transcripts over clinical course and (b) sequence of *ABL1* KD.

**Figure 2:**
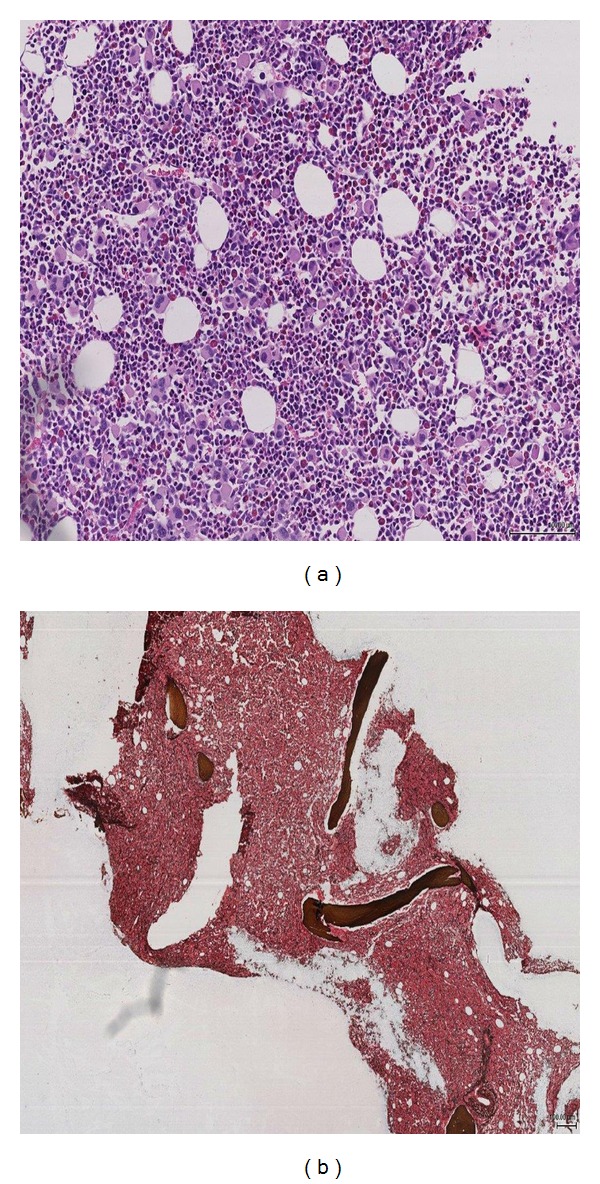
Trephine biopsy at BC progression showing (a) significant megakaryocytic hyperplasia and (b) grade II reticulin fibrosis.

A seventy-eight-year-old male presented with tiredness. He was noted to have raised white cell count and platelets on routine monitoring of his chronic disorders which included hypertension, ischemic heart disease, haemochromatosis, and intracranial aneurysm. A full blood count showed a white cell count 33.5 × 10^9^/L, haemoglobin 13.4 g/dL, and platelets of 762 × 10^9^/L. The white cell differential was comprised of neutrophils 19.8 × 10^9^/L, myelocytes 2.0 × 10^9^/L, metamyelocytes 2.0 × 10^9^/L, lymphocytes 5.0 × 10^9^/L, monocytes 1.3 × 10^9^/L, eosinophils 0.3 × 10^9^/L, and basophils 2.4 × 10^9^/L. A bone marrow aspirate was hypercellular with significant myeloid and megakaryocytic hyperplasia with reduced erythroid series and no fibrosis evident. Cytogenetic analysis revealed 45,X,-Y,t(9; 22)(q34; q11.2) in 20/20 metaphases. A standard reverse-transcriptase polymerase chain reaction (PCR) methodology for detection of *BCR-ABL1* transcripts [18] showed a single band that upon sequencing demonstrated an e19a2 *BCRABL1* fusion leading to a diagnosis of chronic phase CML. The patient commenced on hydroxycarbamide 1 g/day then imatinib 400 mg od one week later. He achieved haematological remission within three months, but thrombocytopenia became rapidly evident prompting an increase in imatinib to 800 mg od. Again, his blood counts normalized, but this was not sustained. Peripheral blood quantitative PCR (qPCR) was performed as previously described [19] at four months and demonstrated no significant decrease in *BCR-ABL1* transcript levels (Figure 1(a)). This prompted investigation for an *ABL1* KD mutation [20]. Sequencing of the *ABL1* KD revealed a Q252H mutation (Figure 1(b)). As *ABL1* KD mutation bearing clones may exist in a minority of CML patients at presentation [21], retrospective analysis was performed but did not demonstrate the presence of the Q252H at presentation. The patient was switched to nilotinib 300 mg bd. At seven months he was deteriorating clinically with increasing splenomegaly and myeloblasts seen in the peripheral blood (white cell count 71.5 × 10^9^/L, basophils 8.6 × 10^9^/L, and myeloblasts 15.0 × 10^9^/L) indicating myeloid blast crisis. A further fragmented bone marrow biopsy demonstrated hypercellularity secondary to increased blast cell numbers (Figure 2(a)) with grade II fibrosis (Figure 2(b)). As the presence of a concurrent myeloproliferative neoplasm (MPN) could have been responsible for the bone marrow fibrosis [22], the most common MPN-associated mutations were retrospectively sought in presentation DNA using an allele-specific PCR approach [23]. No evidence was found of the *JAK2* V617F, *MPL* W515L, or W515 K mutations. The patient commenced nilotinib 300 mg bd with a negligible decrease in *BCR-ABL1* transcripts over the following three months period (Figure 1(a)). He continued to deteriorate clinically, and his final admission was with pyelonephritis due to renal stones. He became increasingly cachectic with worsening splenomegaly and wished to be at home where he died with the cause of death being CML complicated by ischemic heart disease.

## 3. Discussion

The e19a2 is the most common *BCR-ABL1 *variant in CML with patients expressing this transcript type generally considered to respond favourably to TKI therapy. Very few cases of acquired resistance due to *ABL1* KD mutations have been reported in this CML genotype with the Y253H, E355G, T315I, and G250E mutations reported [12–15]. All of these cases achieved significant cytogenetic and/or molecular responses after the introduction of a second generation TKI. This case therefore represents the first description of a Q252H in a patient with e19a2 *BCR-ABL1* CML and who appeared resistant to second generation TKI therapy. Prior to the development of second generation TKIs, the Q252H mutation and further mutations of the ATP phosphate-binding loop (P-loop) of the *ABL1* KD were considered to have a high degree of imatinib resistance and a particularly poor prognosis [24]. However, from subsequent *in vitro* analysis of many *ABL1* KD mutations that has provided a rationale for selection of second generation TKI therapy, the Q252H mutation appears responsive to nilotinib, dasatinib, and bosutinib [25–28]. The lack of response to nilotinib in the case described herein most likely represents the acquisition of further genetic and/or epigenetic events that occur during BC transformation of CML [29].

Also of note is the manifestation of fibrosis associated with the blast crisis. Whereas progressive bone marrow fibrosis is considered an indicator of treatment failure in CML, TKI therapy can reverse fibrosis significantly but does not eliminate its unfavourable prognosis nor guarantee against further fibrotic evolution [30–33]. The fibrosis in this case appeared rapidly in conjunction with progression to blast crisis, a feature previously described in only rare cases [34, 35]. 

This report describes for the first time, the presence of the Q252H mutation in e19a2 *BCR-ABL1* CML and its association with TKI resistance and progression to BC. The case also serves to highlight the considerable challenges in the treatment of CML BC particularly in the elderly patient with significant comorbidities.
